# A prospective study of fatigue trajectories among in‐centre haemodialysis patients

**DOI:** 10.1111/bjhp.12395

**Published:** 2019-11-19

**Authors:** Federica Picariello, Sam Norton, Rona Moss‐Morris, Iain C Macdougall, Joseph Chilcot

**Affiliations:** ^1^ Health Psychology Section, Psychology Department Institute of Psychiatry, Psychology and Neuroscience King’s College London UK; ^2^ Department of Renal Medicine King's College Hospital London UK

**Keywords:** dialysis, fatigue, growth modelling, longitudinal, psychological, vitality

## Abstract

**Objectives:**

Fatigue is common and debilitating among dialysis patients. The aim of this study was to understand the longitudinal trajectory of fatigue and consider sociodemographic, clinical, and psychological factors that are related to variation in fatigue levels over time.

**Design:**

A prospective study of fatigue with yearly assessments over 3 years among prevalent in‐centre haemodialysis (HD) patients.

**Methods:**

Fatigue severity was measured using the Chalder Fatigue Questionnaire and fatigue‐related functional impairment using the Work and Social Adjustment Scale. The trajectories of fatigue outcomes were examined using piecewise growth models, using length of time on dialysis as time. Sociodemographic, clinical, and psychological predictors of fatigue were assessed using linear growth models, using follow‐up time.

**Results:**

One hundred and seventy‐four prevalent HD patients completed baseline measures, 118 at 12 months, 84 at 24 months, and 66 at 36 months. Fatigue severity scores decreased by 0.15 each year. Fatigue‐related functional impairment increased by 1.17 each year. In adjusted linear growth models, non‐white ethnicity was a significant predictor of lower initial fatigue severity (*B *= −2.95, 95% CI −5.51 to −0.40) and a greater reduction in fatigue severity of 1.60 each year (95% CI 0.35–2.36). A one‐point increase in damage beliefs was associated with a 0.36 increase in fatigue‐related functional impairment each year (95% CI −0.61 to −0.01).

**Conclusion:**

Damage beliefs predicted an increase in fatigue‐related functional impairment over time. However, the data strongly suggested that fatigue outcomes vary by length of time on dialysis.

**Statement of contribution**

***What is already known on this subject?***

- At least 1 in 2 haemodialysis (HD) patients are clinically fatigued.
- Growing evidence is available on the important role of psychological factors in fatigue across chronic conditions.
- The contribution of psychological factors, beyond distress, to fatigue in HD has not been examined to date.

***What does this study add?***

- Ethnicity played a role in the initial level of fatigue severity and over time.
- Damage beliefs predicted an increase in fatigue‐related impairment over time.
- Data strongly suggested that fatigue outcomes vary by length of time on dialysis.

## Background

In most developed countries of the world, including the United Kingdom and the United States, the most common renal replacement therapy (RRT) is haemodialysis (HD), particularly at the start and among patients older than 65 (Hole *et al.*, 2018; MacNeill *et al.*, 2018). Fatigue is among the most common symptoms experienced by dialysis patients, affecting at least half, which often interferes with daily life and functioning (Horigan, 2012). Fatigue is a complex and subjective symptom that has been previously described as a distressing and persistent feeling of physical, emotional, and/or cognitive tiredness not proportional to exertion and not relieved by rest (Cella *et al.*, 1998; Dittner *et al.*, 2004; Ream & Richardson, 1996). There is also evidence to suggest that fatigue may contribute directly and indirectly to adverse clinical outcomes in dialysis patients (Bossola *et al.*, 2015; Jhamb *et al.*, 2009, 2011; Koyama *et al.*, 2010; Picariello *et al.*, 2018).

**Figure 1:**
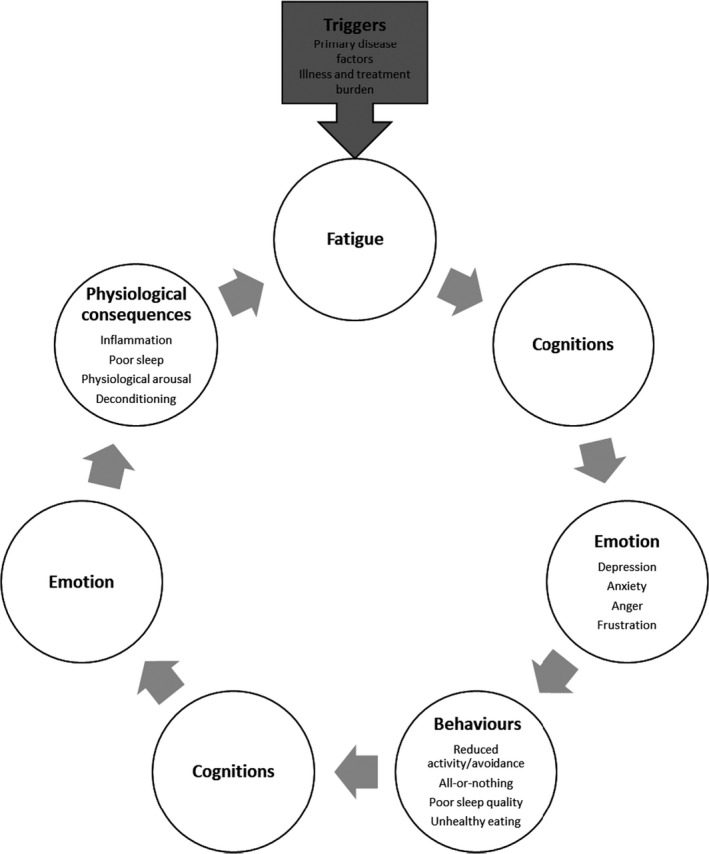
Cognitive‐behavioural model of fatigue in chronic illness. This figure illustrates how fatigue can be triggered by primary disease factors, such as anaemia or haemodialysis in this patient group, and it can then be perpetuated and maintained by a cycle of negative beliefs, depression and/or anxiety, and maladaptive behavioural patterns. Over time, this self‐perpetuating cycle may also lead to physiological consequences, such as increased inflammation, disruption of the sleep–wake cycle, chronic activation of the central and autonomic nervous systems as well as the endocrine system in response to stress, and deconditioning. This can further maintain fatigue and lead to poorer clinical outcomes.

The Standardized Outcomes in Nephrology for HD workshop identified fatigue to be a core outcome (Evangelidis *et al.*, 2017); however, a poor understanding of the pathogenesis of fatigue in this patient population impedes effective fatigue management strategies. Current management of fatigue in the form of pharmacological treatments appears ineffective (Bossola *et al.*, 2011). Exercise‐based interventions, although typically effective (Cheema & Singh, 2005; Segura‐Ortí, 2010), have been criticized for being unsuitable for patients with multimorbidities, disabilities, and in poorer health (Kosmadakis *et al.*, 2010). Therefore, a solely biomedical approach may be insufficient in this setting. An alternative model that has been postulated in the literature to explain fatigue across long‐term conditions (LTCs) is the cognitive‐behavioural model (Wessely *et al.*, 1998). According to this model, primary disease factors, such as anaemia, inflammation, and dialysis may trigger fatigue, yet, how patients react cognitively, emotionally, and behaviourally to the initial symptom of fatigue may worsen, maintain and perpetuate fatigue over time (Figure 1; Donovan *et al.*, 2007; van Kessel & Moss‐Morris, 2006). For example, once fatigue develops as a consequence of underlying disease processes, interpretations of fatigue as uncontrollable and lasting, and unhelpful thinking styles in response to fatigue, such as catastrophizing, symptom focusing, or perceiving fatigue as a sign of bodily damage (damage beliefs), may lead to increased anxiety and low mood and subsequently unhelpful behavioural responses, such as excessive rest in an attempt to control fatigue and reduce the damage perceived it is doing to their body. Excessive rest may in turn lead to deconditioning, poor sleep and physiological arousal related to anxious mood, and lead to the perpetuation of fatigue (van Kessel & Moss‐Morris, 2006; Van der Werf *et al.*, 2003; Vercoulen *et al.*, 1998; Wessely *et al.*, 1998). The importance of these factors in maintaining and perpetuating fatigue has been extensively documented in other conditions, including multiple sclerosis (MS), rheumatoid arthritis (RA), and cancer (Ali *et al.*, 2017; Alsén *et al.*, 2010; Grayson *et al.*, 2013; Jopson & Moss‐Morris, 2003; van Kessel & Moss‐Morris, 2006; Zalai *et al.*, 2015). This conceptualization of fatigue has led to psychological treatments associated with clinically significant improvements in fatigue and related outcomes in MS, RA and cancer (Cramp *et al.*, 2013; Kangas *et al.*, 2008; van den Akker *et al.*, 2016).

Nonetheless, psychological factors remain largely unexplored in fatigue among kidney failure patients (Picariello *et al.*, 2017, 2017b). According to a recent study that examined changes in fatigue over 1 year among HD patients, the mean level of fatigue severity was relatively stable over time, but variations were evident in fatigue status among patients (Bossola *et al.*, 2017), possibly indicative of distinct fatigue trajectories. These variations in status were largely unexplained by sociodemographic and clinical factors, except for the role of comorbidities, but psychological factors were not examined (Bossola *et al.*, 2017). The cross‐sectional data here suggested that dialysis patients’ beliefs, mood, and behaviours contribute to fatigue, in particular, distress, negative beliefs about fatigue, and unhelpful behaviours (all‐or‐nothing and avoidance behaviours) were significant predictors of greater fatigue severity, explaining an additional 36% of variance (Chilcot *et al.*, 2016). Similarly, distress, damage beliefs, avoidance behaviours, and fatigue severity were significant predictors of greater fatigue‐related functional impairment, explaining an additional 44% of variance (Chilcot *et al.*, 2016). In the current paper, the longitudinal findings are presented.

The overarching aim of this study was to understand the longitudinal trajectory of fatigue in HD patients and consider factors that are related to variation in fatigue levels over time, with the following objectives:

*Primary objectives*

1. To model the longitudinal trajectory of fatigue symptoms in dialysis patients over a 3‐year period.

- 2. To evaluate the contribution of demographic, clinical, cognitive, behavioural, and emotional factors to fatigue over a 3‐year period.

*Secondary objective*

- 3. To determine the level and trajectory of functional impairment caused by fatigue over a 3‐year period

Given the paucity of longitudinal fatigue data, an inductive approach to the modelling of trajectories was adopted. We hypothesized that negative beliefs about fatigue, distress, unhelpful cognitive and behavioural responses to fatigue will predict greater fatigue severity and fatigue‐related functional impairment over time, after controlling for sociodemographic and clinical factors.

## Methods

### Study design

This was a prospective study assessing the trajectories and predictors of fatigue severity and fatigue‐related functional impairment over 36 months. Assessments were conducted at yearly intervals (baseline, 12, 24, and 36 months). Ethical approval was granted by East Midlands‐Leicester NRES committee (REC Reference number 14/EM/0037). Patient consent was obtained.

### Participants

Patients were recruited from a renal outpatient service in England. All the patients were receiving thrice‐weekly HD, with sessions generally lasting between 3.5 and 5 hr and relying on exclusively high flux membranes (polysulphone) with bicarbonate used as a buffer. The study ran between April 2014 and August 2017. A total of 279 patients were approached for study participation, with 174 providing informed consent and completing the baseline measures (62.4%). Details of the study including patient recruitment have previously been reported (Chilcot *et al.*, 2016), and further detail on patient attrition is available elsewhere (Picariello *et al.*, 2018). In brief, at 12 months 118 patients (87% of patients still alive, on dialysis, and able to complete the questionnaires) completed questionnaires, at 24 months 84 patients (98% of patients still alive, on dialysis, and able to complete the questionnaires) completed questionnaires, and at 36 months 66 patients completed questionnaires (99% of patients still alive, on dialysis, and able to complete the questionnaires). Although, at 36 months, only 37.9% of the baseline sample was available, attrition was driven by mortality and transplantation, with prevalence rates in line with the renal registry data (Byrne *et al.*, 2018).

The inclusion criteria were as follows: (1) adults with a confirmed kidney failure diagnosis, (2) aged 18 or older, (3) treated with conventional thrice‐weekly in‐centre HD, (4) with a dialysis longevity >90 days, (5) able to speak or write in English, and (6) able and willing to provide informed consent. Patients were excluded if they had significant visual or physical impairment preventing completion of the questionnaires, had any known cognitive impairments, and had serious mental health conditions as noted in their medical history. Patients were not approached if they were judged to be unsuitable by the nursing staff, such as approaching end of life care, repeatedly unwell during the recruitment period or in the process of moving to peritoneal dialysis.

### Data collection

Participants completed the study measures during a stable dialysis session at least 20 min after the initiation of treatment. Further detail on the sociodemographic and clinical data collected at baseline and at each data collection time‐point is available elsewhere (Chilcot *et al.*, 2016). Comorbidity was evaluated using the Charlson Comorbidity Index (CCI) at baseline (Charlson *et al.*, 1987).

#### Psychological measures

All psychological measures were administered at each follow‐up. The primary outcome variable, fatigue severity was measured using the Chalder Fatigue Questionnaire (CFQ; Chalder *et al.*, 1993). This questionnaire has not been developed specifically for HD; therefore, it captures general fatigue. This scale consists of 11 items and scores are assigned for each response, using continuous scoring from 0 to 3. Higher scores represent greater fatigue severity. A cut‐off of greater than 18 defines a fatigue case (Chalder *et al.*, 1993; White *et al.*, 2011). The measure has good psychometric properties in HD patients (Picariello *et al.*, 2016).

The secondary outcome variable, fatigue‐related functional impairment, was measured using the Work and Social Adjustment Scale (WSAS; Mundt *et al.*, 2002). The following psychological predictor variables were measured using validated measures: fatigue perceptions using the Brief Illness Perceptions Questionnaire (BIPQ; Broadbent *et al.*, 2006), cognitive and behavioural responses to fatigue via the Cognitive and Behavioural Responses to Symptoms Questionnaire (CBSQ; Ryan *et al.*, 2018), and distress using the Hospital Anxiety and Depression Scale (HADS; Zigmond & Snaith, 1983). These measures have been previously described in detail (Chilcot *et al.*, 2016). These measures demonstrated acceptable to good psychometric properties (i.e., internal reliabilities >0.70) within the current study.

### Statistical analysis

Questionnaire scores were computed using proration to account for missing items within scales (Graham, 2009). This is described in more detail elsewhere (Picariello *et al.*, 2018). In most cases, single items of a scale were left unanswered with the proportion of item‐level missing data per variable without proration fluctuating between 6.3 and 14.9% at baseline, 3.4 and 7.6% at 12 months, 0 and 2.4% at 24 months, and 0 and 1.5% at 36 months. Given the small volume of missing item data and no systematic patterns of missingness, it is unlikely that multiple imputation at the item level would result in different findings compared to proration, despite criticisms in relation to proration (Mazza *et al.*, 2015).

Approximately 33.3 and 36.6% of observations were missing and not due to death or transplantation in the fatigue severity and fatigue‐related functional impairment models, respectively. Based on descriptive statistics and tabulated patterns, data were unlikely to be missing completely at random, particularly in the comparison of participants who died versus those who were transplanted. A survival analysis from this study cohort indicated that fatigue severity and fatigue‐related functional impairment were significantly associated with an increased risk of death and decreased likelihood of transplantation (Picariello *et al.*, 2018). Nonetheless, Little’s MCAR test at each time‐point indicated that the pattern of missing values did not depend on the data values (baseline χ^2^(*df* = 275, *N* = 174) = 274.59, *p* = .51; 12 months χ^2^(*df* = 178, *N* = 118) = 182.60, *p* = .39; 24 months χ^2^(*df* = 46, *N* = 84) = 49.60, *p* = .33; 36 months χ^2^(*df* = 46, *N* = 66) = 47.79, *p* = .40). Therefore, multiple imputation analyses were conducted, according to Sterne *et al. *(2009) using 20 imputations and including variables in the model associated with missingness, such as total time in the study, and censoring indicators. Multiply imputed data were deleted for patients after they had died or had a transplant; therefore, data were only imputed for 23% of the total sample. This dataset was used for the linear growth models.

Univariate associations between sociodemographic, clinical, and psychological variables with fatigue severity and fatigue‐related functional impairment were examined using bivariate correlations (Pearson or Spearman depending on normal distribution of the data) for continuous variables; ANOVA comparisons for normally distributed categorical variables or the nonparametric Kruskal–Wallis H test. Gender, age, haemoglobin, and dialysis vintage, referring to length of time on dialysis, were included in all models, irrespective of their univariate associations with fatigue outcomes. Although significant correlations were evident between all psychological predictor variables and fatigue outcomes, they were selected for inclusion in the models based on univariate linear growth models.

Fatigue over time was modelled using mixed‐effects regression models, which when applied to longitudinal data are often referred to as linear growth models (Rabe‐Hesketh & Skrondal, 2008; StataCorp Longitudinal, 2005). Fatigue severity and fatigue‐related functional impairment were used as continuous outcome variables in the models. These models are a generalization of the standard linear regression model that allow the analysis of repeated measures through the inclusion of *random effects*, which are estimates of the between‐person variability. In the analyses presented here, we allowed for both a *random intercept*, capturing variability in the initial level of fatigue, and a *random slope* for the time variable, capturing variability in the rate of change in fatigue. Here, time was coded as months from the baseline assessment. Non‐linear functions of time were assessed; however, standard criteria (chi‐square and fit indices) indicated a linear rate of change as providing the most parsimonious fit to the data. The association between hypothesized predictors with the initial level of fatigue and the rate of change in fatigue was assessed by including a predictor variable and a predictor by time interaction term in the model.

Between‐person and within‐person variability was considered as part of exploratory data analysis, including an exploration of any time‐dependent effects of age and dialysis vintage based on the mixed evidence on the association of these constructs with fatigue in this patient population. Exploratory analysis indicated that both fatigue severity and fatigue‐related functional impairment varied non‐linearly by dialysis vintage. The identified pattern in fatigue severity also makes sense physiologically, as well as psychologically, as patients in pre‐dialysis care have an accumulation of fluid and toxins in their bodies, which improves with initiation of dialysis, but over time, the physiological consequences of dialysis and treatment burden may contribute to the increase in fatigue. Therefore, an alternative parameterization of time to the one above was implemented where time was coded as months from dialysis initiation, rather than time from baseline assessment. A piecewise linear growth model was estimated. This model extends the model described above to allow for different phases of change by the inclusion of more than one random time slope (Bryk & Raudenbush, 1992; Van Der Leeden, 1998). Specifically, we included two linear time slopes with a change point at 24 months allowing for the estimation of the initial level of fatigue, the rate of change in fatigue between 0 and 24 months from dialysis initiation, and the rate of change after 24 months from dialysis initiation. Predictors of fatigue outcomes were examined using linear growth models described earlier because more than half of the sample was receiving dialysis for 24 months or longer at baseline (*N* = 101, 58%).

Descriptive statistics and exploratory statistics were conducted in SPSS version 24. Models looking at fatigue over time and predictors of fatigue outcomes were conducted in STATA version 15. Data were converted from wide to long format and set as longitudinal (*xtset*) (Torres‐Reyna, 2007) to use the *mixed* command for multilevel mixed‐effects linear regressions (Box 1; Rabe‐Hesketh & Skrondal, 2008; StataCorp Longitudinal, 2005). To apply the piecewise model, dialysis vintage was recoded to fit linear models in each period (Bryk & Raudenbush, 1992), where at each follow‐up for slope 1 dialysis vintage of over 24 months was capped at 24, whereas for slope 2, dialysis vintage of 24 months or shorter was recoded as 0. Significance was set at *p* ≤ .05, using the standard α cut‐off.

Box 1 **STATA commands used**

**Table:** 

Piecewise growth model command
*mi estimate: mixed CFQ/WSAS dialysisvintage_1_ dialysisvintage_2_ || id:*
Linear growth model using follow‐up time with predictors command
*mi estimate: mixed CFQ/WSAS(c.time)##(list of predictors) || id:*

## Results

### Sample characteristics

Baseline sociodemographic and clinical characteristics have been previously described elsewhere (Chilcot *et al.*, 2016) and are available in Table 1. Most of the sample was male (63.2%) with a mean age of 59 years old (*SD* = 15.17). The median dialysis vintage (time on dialysis) was 35 months (interquartile range: 52). A bar chart summarizing the frequency distribution of dialysis vintage at baseline in this sample can be found in the Supplementary File S1. At baseline, mean fatigue was 17.34 (*SD* = 6.54) and 82 patients (47.1%) scored above the threshold for clinical fatigue, scoring 18 or above on the CFQ. Fatigue severity ranged from 16.49 (*SD* = 6.12 at 36 months) to 18.26 (*SD* = 6.27 at 24 months) across follow‐up time‐points. At baseline, the mean fatigue‐related functional impairment score was 18.5 (*SD* = 13.00), reaching a maximum of 22.37 (*SD* = 11.44) at 24 months. Descriptive fatigue data over time are presented in Table 2.

**Table 1:** Sociodemographic, clinical, and biochemical characteristics of the sample at baseline (*n* = 174)

Variable	Statistic
Sociodemographic characteristics	
Female (*N*, %)	64 (36.8%)
Age (*M*, *SD*, range)	59 (*SD* = 15.17); range = 25–92
BMI (*M*, *SD*, range)	28.33 (*SD* = 6.21); range = 17.40–49.80
Ethnicity (*N*, %)	
Caucasian	75 (43.1%)
Black	81 (46.6%)
Asian	15 (8.6%)
Marital status (*N*, %)	
Married/living with partner	78 (44.8%)
Divorced/separated/never married/single/single parent	72 (41.4%)
Living with partner, friends, relatives	94 (54.0%)
Living alone	48 (27.6%)
Employment status (*N*, %)	
Working full‐time/working part‐time/housekeeping/ self‐employed	29 (16.7%)
Retired	59 (33.9%)
Unemployed	14 (8.0%)
None of the above	7 (4.0%)
Smoking status (*N*, %)	
Smoker	18 (10.3%)
Ex‐smoker	45 (25.9%)
Non‐smoker	111 (63.8%)
Exercise status (*N*, %)	
More than three times per week	44 (25.3%)
Less than three times per week	51 (29.3%)
Not at all	79 (45.4%)
Clinical characteristics	
Transplant status (*N*, %)	
Active	33 (19.0%)
Unfit but to reconsider/suspended/being worked up for transplant	75 (43.1%)
Unfit/ unfit permanently/patient requested	65 (37.4%)
Primary renal diagnosis (*N*, %)	
Type 2 diabetes related	45 (25.9%)
Hypertensive renal failure	41 (23.6%)
Access type (*N*, %)	
Haemocath	47 (27.0%)
Brachial fistula	83 (47.7%)
Radial fistula	35 (20.1%)
Arm	9 (5.2%)
Dialysis vintage in months (median, interquartile range, range)	35 (interquartile range = 52); range = 3–304
ESA (*N*, %)	
Yes	141 (81%)
No	9 (5.2%)
I don’t know	21 (12.1%)
Charlson Comorbidity Score (*M*, *SD*, range)	3.85 (*SD* = 1.93); range = 2–9
Biochemical characteristics	
Haemoglobin (g/L) (*M*, *SD*, range)	109.56 (*SD* = 11.09); range = 62–140
Albumin (g/L) (*M*, *SD*, range)	39.33 (*SD* = 3.96); range = 22–55
Creatinine (µmol/L) (*M*, *SD*, range)	809.53 (*SD* = 292.34); range = 182–1,567
Urea (mmol/L) (*M*, *SD*, range)	20.10 (*SD* = 6.95); range = 4–57
IDWL (kg) (*M*, *SD*, range)	−1.91 (*SD* = 1.01); range = −6.40 to −0.10
URR (%) (*M*, *SD*, range)	67.61 (*SD* = 6.93); range = 31–91
CRP (mg/L) (median, interquartile range, range)	11.50 (interquartile range = 20.90); range = 1–216

Notes

BMI = body mass index; ESA = erythropoietin‐stimulating agents; IDWL = intradialytic weight loss (weight lost during a dialysis session); URR = urea reduction ratio (marker of dialysis adequacy); CRP = C‐reactive protein (marker of inflammation).

**Table 2:** Psychological variables: descriptive statistics over time

Questionnaire	Time‐points								Change over time based on unadjusted linear growth model output
	Baseline (*N* = 174)		12‐month follow‐up (*N* = 118)		24‐month follow‐up (*N* = 84)		36‐month follow‐up (*N* = 66)		
	Response Range	Mean (*SD*)	Response Range	Mean (*SD*)	Response Range	Mean (*SD*)	Response Range	Mean (*SD*)	
CFQ									
Fatigue severity	0–33	17.34 (*SD* = 6.54)	2–33	17.37 (*SD* = 5.80)	1–33	18.26 (*SD* = 6.27)	2–31	16.49 (*SD* = 6.12)	*B *= −0.15, *p* = .46, 95% CI −0.56 to 0.25
WSAS									
Fatigue‐related functional impairment	0–40	18.08 (*SD* = 13.48)	0–40	21.13 (*SD* = 11.97)	0–39	22.37 (*SD* = 11.44)	0–40	21.64 (*SD* = 10.93)	*B* = 1.17, *p* = .002, 95% CI 0.42 to 1.92†
HADS									
Distress	0–35	13.89 (*SD* = 8.49)	0–35	11.98 (*SD* = 7.69)	0–37	12.52 (*SD* = 9.16)	0–31	13.48 (*SD* = 7.80)	*B*=−0.46, *p* = .038, 95% CI −0.90 to −0.03†
BIPQ (without item A)									
Total illness perception	0–60	30.81 (*SD* = 13.53)	0–55	30.03 (*SD* = 11.71)	0–60	29.87 (*SD* = 12.53)	8–53	30.45 (*SD* = 9.26)	*B*=−0.21, *p* = .70, 95% CI −1.27 to 0.85
CBSQ									
Fear avoidance	0–23	10.99 (*SD* = 4.48)	1–24	10.71 (*SD* = 4.45)	1–24	12.36 (*SD* = 5.68)	0–23	11.52 (*SD* = 4.93)	*B* = 0.31, *p* = .09, 95% CI −0.13 to 0.85
Catastrophizing	0–16	7.03 (*SD* = 3.91)	0–16	6.94 (*SD* = 3.93)	0–15	6.19 (*SD* = 3.58)	0–16	7.05 (*SD* = 3.89)	*B*=−0.13, *p* = .52, 95% CI −0.05 to 0.68
Symptom focusing	0–24	11.40 (*SD* = 5.66)	0–24	11.09 (*SD* = 6.15)	0–24	9.67 (*SD* = 7.44)	0–24	11.77 (*SD* = 6.99)	*B*=−0.31, *p* = .16, 95% CI −0.74 to 0.12
Damage beliefs	0–20	10.11 (*SD* = 3.60)	0–18	10.02 (*SD* = 4.04)	0–19	9.83 (*SD* = 5.01)	0–19	10.17 (*SD* = 4.12)	*B*=−0.10, *p* = .52, 95% CI −0.40 to 0.20
Embarrassment avoidance	0–24	8.33 (*SD* = 5.77)	0–23	8.26 (*SD* = 5.78)	0–24	7.45 (*SD* = 6.57)	0–24	9.59 (*SD* = 6.57)	*B* = 0.05, *p* = .83, 95% CI −0.37 to 0.46
All‐or‐nothing behaviours	0–20	6.16 (*SD* = 4.71)	0–20	5.89 (*SD* = 4.73)	0–20	7.50 (*SD* = 5.56)	0–18	6.70 (*SD* = 4.45)	*B* = 0.24, *p* = .16, 95% CI −0.09 to 0.57
Avoidance behaviours	0–32	12.28 (*SD* = 7.12)	0–29	11.60 (*SD* = 6.77)	2–30	14.01 (*SD* = 7.19)	0–29	12.49 (*SD* = 7.81)	*B* = 0.16, *p* = .52, 95% CI −0.32 to 0.63

† *p* < .05.

Just over half of the participants who were fatigued at baseline remained fatigued at 12 months and slightly less than a quarter of participants remained fatigued by 36 months. Around 9 to 14% of participants who were not fatigued at baseline became fatigued at follow‐up.

### Psychological variables over time

Table 2 displays the descriptive characteristics of the psychological variables at each time‐point, based on the observed data. According to linear growth models, the yearly change in fatigue severity was −0.15 (*p* = .46, 95% CI −0.56 to 0.25), while there was a significant increase in fatigue‐related functional impairment of 1.17 each year (*p* = .002, 95% CI 0.42 to 1.92). Using the MI dataset, the yearly change in fatigue severity was −0.11 (*p* = .68, 95% CI −0.63 to 0.41) and in fatigue‐related functional impairment was 1.40 (*p* = .010, 95% CI 0.33 to 2.46). Similarly, relying only on participants who completed each follow‐up (*N* = 66), the yearly change in fatigue severity was −0.24 (*p* = .31, 95% CI −0.71 to 0.23) and in fatigue‐related functional impairment was 0.91 (*p* = .037, 95% CI 0.06 to 1.75). Using the MI dataset, without participants who died or were transplanted over the course of the study; produced similar results, with a yearly change in fatigue severity of −0.13 (*p* = .68, 95% CI −0.75 to 0.49) and in fatigue‐related functional impairment of 1.39 (*p* = .041, 95% CI 0.06 to 2.73). Therefore, the findings are generally congruent.

Based on unadjusted linear growth models, there were no significant changes over time in any of the psychological predictor variables, except for distress (Table 2). The yearly reduction in distress was −0.46 (*p* = .038, 95% CI −0.90 to −0.03).

### Predictors of fatigue severity over time

The results for each step of the model are available in Table 3. In the final model, including all the predictor variables (*N* = 14), only ethnicity was a significant predictor of the initial level of fatigue severity (*B *= −2.95, *p* = .024, 95% CI −5.51 to −0.40) and change in fatigue severity (*B* = 1.36, *p* = .009, 95% CI 0.35 to 2.36). Non‐white ethnicity was associated with a 2.95 lower initial fatigue severity and a reduction in fatigue severity of 1.60 points each year. These coefficients represent clinically significant changes in fatigue severity on the CFQ, particularly taking into consideration the additive impact of ethnicity over time (Nordin *et al.*, 2016). In addition, a one‐point increase in haemoglobin was predictive of a small, but not clinically meaningful (Nordin *et al.*, 2016), increase in fatigue severity of .02 each year (*B *= −0.05, *p* = .050, 95% CI −0.10, −0.00008).

**Table 3:** Predictors of fatigue severity (CFQ) over time

Step and variable	Initial level of fatigue			Rate of change in fatigue	
	*B* (95% CI)	Significance level (2‐tailed)	Slope	*B* (95% CI)	Significance level (2‐tailed)
1					
Gender	.58 (−1.71, 2.87)	0.62	−1.50	.16 (−0.89, 1.20)	0.77
Age (time‐dependent)	−.02 (−0.10, 0.05)	0.55		.01 (−0.02, 0.05)	0.47
Ethnicity	**−2.30 (−4.47, −0.13)**	**0.038**		**1.13 (0.09, 2.17)**	**0.033**
Employment status (at baseline)	−2.61 (−5.70, 0.49)	0.10		.41 (−0.92, 1.74)	0.55
BMI (at baseline)	.18 (−0.03, 0.40)	0.09		−.03 (−0.11, 0.06)	0.56
Exercise status (at baseline)	**−2.95 (−5.24, −0.65)**	**0.012**		.96 (−0.06, 1.97)	0.07
2					
Gender	.42 (−1.77, 2.61)	0.71	2.60	.16 (−0.86, 1.19)	0.75
Age (time‐dependent)	−.04 (−0.12, 0.04)	0.30		.02 (−0.02, 0.05)	0.32
Ethnicity	**−2.58 (−4.72, −0.43)**	**0.019**		**1.22 (0.18, 2.25)**	**0.021**
Employment status (at baseline)	−2.26 (−5.28, 0.75)	0.14		.43 (−0.89, 1.74)	0.53
BMI (at baseline)	.13 (−0.09, 0.35)	0.24		−.01 (−0.10, 0.08)	0.83
Exercise status (at baseline)	**−3.21 (−5.42, −.99)**	**0.005**		**1.08 (0.05, 2.10)**	**0.039**
CCI (at baseline)	.59 (−0.003, 1.18)	0.05		−.13 (−0.39, 0.14)	0.36
Dialysis vintage (time‐dependent)	.009 (−0.02, 0.04)	0.54		.003 (−0.007, 0.01)	0.54
Haemoglobin (time‐dependent)	.05 (−.11, 0.22)	0.51		−.04 (−0.10, 0.009)	0.11
3					
Gender	.39 (−1.67, 2.46)	0.71	4.21	.07 (−0.93, 1.07)	0.89
Age (time‐dependent)	−.01 (−0.08, 0.06)	0.78		.01 (−0.02, 0.05)	0.48
Ethnicity	**−2.92 (−4.99,** **−0.86)**	**0.006**		**1.32 (0.30, 2.33)**	**0.011**
Employment status (at baseline)	−1.47 (−4.18, 1.23)	0.28		.13 (−1.13, 1.40)	0.84
BMI (at baseline)	.06 (−0.16, 0.27)	0.60		.009 (−0.08, 0.10)	0.84
Exercise status (at baseline)	−2.20 (−4.53, 0.13)	0.06		.79 (−0.23, 1.81)	0.13
CCI (at baseline)	.41 (−0.15, 0.97)	0.15		−.11 (−0.37, 0.15)	0.40
Dialysis vintage (time‐dependent)	.01 (−0.02, 0.04)	0.47		.001 (−0.009, 0.01)	0.79
Haemoglobin (time‐dependent)	.07 (−0.10, 0.25)	0.41		**−.05 (−0.11, −0.001)**	**0.045**
Distress (time‐dependent)	**.30 (0.006, 0.59)**	**0.046**		−.03 (−0.10, 0.04)	0.38
4					
Gender	.39 (−1.55, 2.34)	0.69	4.21	.10 (−0.87, 1.07)	0.84
Age (time‐dependent)	−.003 (−0.07, 0.07)	0.94		.01 (−0.02, 0.04)	0.55
Ethnicity	**−2.80 (−5.01, −**0**.59)**	**0.013**		**1.31 (0.29, 2.33)**	**0.012**
Employment status (at baseline)	−1.24 (−3.88, 1.41)	0.36		.05 (−1.21, 1.31)	0.93
BMI (at baseline)	.04 (−0.18, 0.26)	0.71		.01 (−0.08, 0.10)	0.79
Exercise status (at baseline)	−1.86 (−4.42, 0.70)	0.15		.68 (−0.40, 1.75)	0.22
CCI (at baseline)	.32 (−0.29, 0.93)	0.30		−.08 (−0.36, 0.20)	0.57
Dialysis vintage (time‐dependent)	.008 (−0.02, 0.04)	0.53		.001 (−0.009, 0.01)	0.79
Haemoglobin (time‐dependent)	.08 (−0.08, 0.23)	0.32		**−.05 (−0.10, −0.005)**	**0.029**
Distress (time‐dependent)	.26 (−0.08, 0.61)	0.13		−.03 (−0.12, 0.07)	0.58
Negative beliefs about fatigue (time‐dependent)	.06 (−0.21, 0.33)	0.65		.002 (−0.06, 0.06)	0.96
5					
Gender	.54 (−1.39, 2.47)	0.58	4.98	.002 (−0.95, 0.95)	1.00
Age (time‐dependent)	.0003 (−0.07, 0.07)	0.99		.009 (−0.02, 0.04)	0.58
Ethnicity	**−2.91 (−5.52, −0.29)**	**0.030**		**1.39 (0.37, 2.41)**	**0.008**
Employment status (at baseline)	−.89 (−3.59, 1.81)	0.52		−.09 (−1.34, 1.16)	0.89
BMI (at baseline)	.04 (−0.16, 0.25)	0.68		.01 (−0.07, 0.10)	0.79
Exercise status (at baseline)	−1.74 (−4.17, 0.69)	0.16		.63 (−0.41, 1.66)	0.23
CCI (at baseline)	.30 (−0.31, 0.91)	0.33		−.08 (−0.35, 0.20)	0.59
Dialysis vintage (time‐dependent)	.009 (−0.02, 0.03)	0.46		.001 (−0.008, 0.01)	0.83
Haemoglobin (time‐dependent)	.07 (−0.08, 0.23)	0.33		**−.05 (−0.10, −0.005)**	**0.032**
Distress (time‐dependent)	.18 (−0.18, 0.54)	0.32		.01 (−0.09, 0.12)	0.80
Negative beliefs about fatigue (time‐dependent)	.07 (−0.20, 0.34)	0.59		.0004 (−0.06, 0.06)	0.99
Damage beliefs (time‐dependent)	.16 (−0.57, 0.89)	0.66		−.08 (−0.29, 0.14)	0.47
Embarrassment avoidance (time‐dependent)	.11 (−0.39, 0.60)	0.67		−.05 (−0.19, 0.08)	0.43
6					
Gender	.68 (−1.18, 2.53)	0.47	4.61	−.03 (−0.96, 0.91)	0.95
Age (time‐dependent)	.004 (−0.06, 0.07)	0.92		.01 (−0.02, 0.04)	0.56
Ethnicity	**−2.95 (−5.51, −0.40)**	**0.024**		**1.36 (0.35, 2.36)**	**0.009**
Employment status (at baseline)	−.75 (−3.42, 1.92)	0.58		−.07 (−1.29, 1.15)	0.91
BMI (at baseline)	.05 (−0.15, 0.25)	0.62		.0008 (−0.08, 0.08)	0.98
Exercise status (at baseline)	−1.25 (−3.73, 1.24)	0.32		.43 (−0.61, 1.47)	0.42
CCI (at baseline)	.23 (−0.42, 0.88)	0.49		−.04 (−0.33, 0.24)	0.76
Dialysis vintage (time‐dependent)	.01 (−0.01, 0.03)	0.38		.0008 (−0.008, 0.01)	0.86
Haemoglobin (time‐dependent)	.07 (−0.09, 0.23)	0.37		**−.05 (−0.10, −0.00008)**	**0.050**
Distress (time‐dependent)	.15 (−0.22, 0.51)	0.41		.007 (−0.10, 0.11)	0.89
Negative beliefs about fatigue (time‐dependent)	.06 (−0.21, 0.33)	0.63		.004 (−0.05, 0.06)	0.89
Damage beliefs (time‐dependent)	.13 (−0.59, 0.86)	0.71		−.09 (−0.30, 0.11)	0.38
Embarrassment avoidance (time‐dependent)	.12 (−0.36, 0.60)	0.60		−.07 (−0.21, 0.07)	0.33
Avoidance behaviours (time‐dependent)	.11 (−0.13, 0.36)	0.36		.03 (−0.06, 0.13)	0.53

Notes

Variables were entered into the model based on their univariate association with fatigue outcomes, to account for multiple tests an adjusted *p*‐value cut‐off of *p* = .002 was also used at baseline (Bonferroni correction 0.05/31), but with reduced power over time given attrition, the magnitude of the association was considered (*r* ≤ 0.30) regardless of the *p*‐value.

CI = confidence interval.

Bold values indicate *p* < .05.

Engaging in exercise at baseline was a significant predictor of lower initial fatigue severity, until distress was added to the model. Similarly, exercise status at baseline was predictive of a reduction in fatigue severity of 1.98 (step 1) and 2.13 (step 2) each year, respectively, until distress was added to the model. Distress was a significant predictor of the initial level of fatigue severity, where a one‐point increase in distress was associated with a 0.30 increase in initial fatigue severity. This association ceased to be significant after negative beliefs about fatigue were added to the model. Lastly, a one‐point increase in comorbidities (CCI) was associated with a 0.59 higher initial level of fatigue severity, but this was only marginally significant (95% CI −0.003, 1.18; step 2).

### Predictors of fatigue‐related functional impairment over time

The results for each step of the model are available in Table 4. In the final model, including all the predictor variables (*N* = 15), no significant predictors of the initial level of fatigue‐related functional impairment could be identified. On the contrary, change in fatigue‐related functional impairment was significantly associated with damage beliefs, after controlling for sociodemographic, clinical, and other psychological variables (*B *= −0.31, *p* = .041, 95% CI −0.61 to −0.01). A one‐point increase in damage beliefs was associated with a 0.36 increase in fatigue‐related functional each year. This is unlikely to be a clinically significant change in fatigue‐related functional impairment (Zahra *et al.*, 2014); however, over 3 years this would amount to an increase of 1.08 in fatigue‐related functional impairment, where following therapy the mean change in WSAS has been reported to be −5.07 (Zahra *et al.*, 2014).

**Table 4:** Predictors of fatigue‐related functional impairment (WSAS) over time

Step and variable	Initial level of fatigue			Rate of change in fatigue	
	*B* (95% CI)	Significance level (2‐tailed)	Slope	*B* (95%)	Significance level (2‐tailed)
1					
Gender	.60 (−4.01, 5.21)	0.80	−.11	.62 (−1.44, 2.69)	0.55
Age (time‐dependent)	−.02 (−0.17, 0.13)	0.82		.02 (−0.06, 0.09)	0.67
Employment status (at baseline)	**−9.10 (−15.03, −3.17)**	**0.003**		2.01 (−0.66, 4.68)	0.14
BMI (at baseline)	**.38 (0.008, 0.74)**	**0.045**		−.05 (−0.21, 0.12)	0.56
Exercise status (at baseline)	**−6.73 (−10.98, −2.49)**	**0.002**		**2.31 (0.38, 4.25)**	**0.019**
2					
Gender	−.11 (−4.57, 4.36)	0.96	−2.18	.85 (−1.13, 2.83)	0.40
Age (time‐dependent)	−.09 (−0.24, 0.06)	0.24		.03 (−0.04, 0.10)	0.37
Employment status (at baseline)	**−7.07 (−12.87, −1.27)**	**0.017**		1.39 (−1.30, 4.08)	0.31
BMI (at baseline)	.25 (−0.14, 0.65)	0.21		−.03 (−0.21, 0.14)	0.71
Exercise status (at baseline)	**−6.99 (−11.08, −2.90)**	**0.001**		**2.39 (0.47, 4.30)**	**0.015**
CCI (at baseline)	1.13 (−0.09, 2.35)	0.07		−.06 (−0.58, 0.45)	0.82
Transplant list status (at baseline)	5.11 (−0.09, 10.31)	0.05		−1.98 (−4.38, 0.42)	0.11
Dialysis vintage (time‐dependent)	.03 (−0.03, 0.08)	0.36		−.008 (−0.03, 0.01)	0.47
Haemoglobin (time‐dependent)	−.13 (−0.55, 0.29)	0.53		.02 (−0.08, 0.13)	0.66
3					
Gender	−.10 (−4.32, 4.11)	0.96	2.34	.63 (−1.28, 2.53)	0.52
Age (time‐dependent)	−.01 (−0.17, 0.15)	0.90		.01 (−0.06, 0.08)	0.73
Employment status (at baseline)	−5.49 (−11.21, 0.22)	0.06		.85 (−1.77, 3.48)	0.52
BMI (at baseline)	.09 (−0.30, 0.49)	0.64		.01 (−0.16, 0.18)	0.90
Exercise status (at baseline)	**−4.63 (−8.60, −0.65)**	**0.023**		1.68 (−0.21, 3.57)	0.08
CCI (at baseline)	.73 (−0.37, 1.83)	0.19		−.01 (−0.50, 0.47)	0.95
Transplant list status (at baseline)	4.01 (−0.92, 8.94)	0.11		−1.56 (−3.87, 0.75)	0.19
Dialysis vintage (time‐dependent)	.03 (−0.03, 0.08)	0.34		−.01 (−0.03, 0.008)	0.25
Haemoglobin (time‐dependent)	−.07 (−0.43, 0.29)	0.70		−.002 (−0.10, 0.10)	0.96
Distress (time‐dependent)	**.69 (0.21, 1.18)**	**0.006**		−.09 (−0.23, 0.04)	0.18
4					
Gender	−.003 (−3.97, 3.96)	1.00	2.89	.61 (−1.25, 2.47)	0.52
Age (time‐dependent)	.004 (−0.13, 0.14)	0.95		.008 (−0.06, 0.07)	0.82
Employment status (at baseline)	−5.13 (−10.66, 0.40)	0.07		.74 (−1.74, 3.22)	0.56
BMI (at baseline)	.06 (−0.30, 0.42)	0.75		.02 (−0.14, 0.18)	0.79
Exercise status (at baseline)	−3.82 (−8.52, 0.88)	0.11		1.38 (−0.66, 3.42)	0.19
CCI (at baseline)	.54 (−0.63, 1.71)	0.36		.06 (−0.47, 0.59)	0.84
Transplant list status (at baseline)	3.84 (−0.94, 8.62)	0.11		−1.54 (−3.81, 0.73)	0.18
Dialysis vintage (time‐dependent)	.02 (−0.03, 0.07)	0.41		−.01 (−0.03, 0.008)	0.28
Haemoglobin (time‐dependent)	−.06 (−0.37, 0.25)	0.69		−.003 (−0.09, 0.09)	0.95
Distress (time‐dependent)	.55 (−0.15, 1.25)	0.12		−0.05 (−0.23, 0.13)	0.60
Negative beliefs about fatigue (time‐dependent)	.18 (−0.38, 0.73)	0.51		−0.04 (−0.16, 0.08)	0.51
5					
Gender	.51 (−2.94, 3.96)	0.77	6.38	.47 (−1.26, 2.21)	0.59
Age (time‐dependent)	.02 (−0.12, 0.15)	0.81		.0003 (−0.06, 0.06)	0.99
Employment status (at baseline)	−4.13 (−9.54, 1.27)	0.13		.44 (−1.92, 2.79)	0.72
BMI (at baseline)	.05 (−0.30, 0.40)	0.76		.02 (−0.13, 0.18)	0.77
Exercise status (at baseline)	−3.45 (−8.07, 1.17)	0.14		1.13 (−0.86, 3.12)	0.26
CCI (at baseline)	.44 (−0.68, 1.57)	0.44		.10 (−0.41, 0.60)	0.71
Transplant list status (at baseline)	3.96 (−0.48, 8.41)	0.08		−1.57 (−3.70, 0.56)	0.15
Dialysis vintage (time‐dependent)	.02 (−0.02, 0.07)	0.28		−.01 (−0.03, 0.005)	0.17
Haemoglobin (time‐dependent)	−.05 (−0.35, 0.25)	0.72		−.009 (−0.10, 0.08)	0.85
Distress (time‐dependent)	.38 (−0.50, 1.25)	0.38		−.02 (−0.25, 0.21)	0.87
Negative beliefs about fatigue (time‐dependent)	.17 (−0.34, 0.69)	0.49		−.04 (−0.16, 0.07)	0.43
Damage beliefs (time‐dependent)	.79 (−0.27, 1.86)	0.14		−.33 (−0.66, 0.006)	0.05
Embarrassment avoidance (time‐dependent)	.08 (−0.93, 1.09)	0.87		.09 (−0.16, 0.35)	0.47
6					
Gender	.82 (−2.66, 4.31)	0.64	6.09	.38 (−1.32, 2.08)	0.66
Age (time‐dependent)	.03 (−0.11, 0.16)	0.69		−.002 (−0.06, 0.06)	0.94
Employment status (at baseline)	−3.87 (−8.85, 1.11)	0.13		.36 (−1.95, 1.68)	0.76
BMI (at baseline)	.07 (−0.26, 0.40)	0.68		.01 (−0.14, 0.16)	0.87
Exercise status (at baseline)	−2.29 (−6.88, 2.29)	0.32		.69 (−1.26, 2.65)	0.49
CCI (at baseline)	.29 (−0.78, 1.36)	0.59		.15 (−0.35, 0.65)	0.55
Transplant list status (at baseline)	3.67 (−0.60, 7.94)	0.09		−1.45 (−3.49, 0.60)	0.17
Dialysis vintage (time‐dependent)	.03 (−0.02, 0.07)	0.21		−.01 (−0.03, 0.004)	0.12
Haemoglobin (time‐dependent)	−.05 (−0.31, 0.20)	0.67		−.0007 (−0.09, 0.09)	0.99
Distress (time‐dependent)	.28 (−0.46, 1.03)	0.44		.003 (−0.20, 0.21)	0.98
Negative beliefs about fatigue (time‐dependent)	.15 (−0.33, 0.63)	0.52		−.03 (−0.14, 0.08)	0.54
Damage beliefs (time‐dependent)	.75 (−0.34, 1.83)	0.17		**−.34 (−0.65, −0.03)**	**0.034**
Embarrassment avoidance (time‐dependent)	.04 (−0.90, 0.98)	0.93		.11 (−0.14, 0.36)	0.39
Avoidance behaviours (time‐dependent)	.35 (−0.34, 1.04)	0.31		−0.07 (−0.23, 0.10)	0.42
7					
Gender	.53 (−2.76, 3.83)	0.75	3.87	.39 (−1.23, 2.01)	0.64
Age (time‐dependent)	.02 (−0.10, 0.15)	0.71		−.003 (−0.06, 0.06)	0.92
Employment status (at baseline)	−3.44 (−8.24, 1.35)	0.16		.28 (−1.97, 2.52)	0.81
BMI (at baseline)	.04 (−0.28, 0.36)	0.82		.02 (−0.13, 0.17)	0.80
Exercise status (at baseline)	−1.67 (−5.44, 2.10)	0.38		.48 (−1.29, 2.26)	0.59
CCI (at baseline)	.21 (−0.75, 1.17)	0.66		.16 (−0.30, 0.62)	0.48
Transplant list status (at baseline)	2.91 (−1.38, 7.20)	0.18		−1.35 (−3.33, 0.62)	0.18
Dialysis vintage (time‐dependent)	.02 (−0.02, 0.06)	0.29		−.01 (−0.03, 0.003)	0.11
Haemoglobin (time‐dependent)	−.08 (−0.32, 0.15)	0.47		.02 (−0.07, 0.10)	0.66
Distress (time‐dependent)	.22 (−0.48, 0.92)	0.52		−.0008 (−0.19, 0.19)	0.99
Negative beliefs about fatigue (time‐dependent)	.12 (−0.28, 0.52)	0.54		−.03 (−0.14, 0.09)	0.65
Damage beliefs (time‐dependent)	.67 (−0.25, 1.58)	0.15		**−.31 (−0.61, −0.01)**	**0.041**
Embarrassment avoidance (time‐dependent)	−.02 (−0.87, 0.83)	0.96		.14 (−0.09, 0.38)	0.23
Avoidance behaviours (time‐dependent)	.29 (−0.40, 0.98)	0.40		−.07 (−0.23, 0.09)	0.40
Fatigue severity (time‐dependent)	.51 (−0.18, 1.21)	0.14		−.04 (−0.23, 0.16)	0.69

Notes

Variables were entered into the model based on their univariate association with fatigue outcomes, to account for multiple tests an adjusted *p*‐value cut‐off of *p* = .002 was also used at baseline (Bonferroni correction 0.05/31), but with reduced power over time given attrition, the magnitude of the association was considered (*r* ≤ 0.30) regardless of the *p*‐value. CI = confidence interval.

Bold values indicate *p* < .05.

Higher BMI at baseline was a significant predictor of greater initial fatigue‐related functional impairment (step 1), yet ceased to be significant when clinical factors were added to the model. Being employed and engaging in exercise at baseline, on the other hand, were significant predictors of lower initial fatigue‐related functional impairment. However, employment status was only marginally significant after distress was added to the model, while exercise status remained a significant predictor of initial fatigue‐related functional impairment until negative beliefs about fatigue were added to the model. Additionally, in steps 1 and 2, engaging in exercise at baseline was predictive of a reduction in fatigue‐related functional impairment by 1.19 each year, until distress was added to the model.

Being deemed unfit for a transplant at baseline was also predictive of greater initial fatigue‐related functional impairment in step 2, but this association was only marginally significant (95% CI −0.09 to 10.31). Lastly, a one‐point increase in distress was associated with a 0.69 increase in initial fatigue‐related functional impairment, but this association ceased to be significant after negative beliefs about fatigue were added to the model.

The predictors of fatigue severity and fatigue‐related functional impairment were generally consistent when including only participants who self‐identified as White, Black African, Black Caribbean, or Black Other (*N* = 156; please see Supplementary File S4).

### Fatigue piecewise growth models

According to a piecewise growth model based on the observed data, in the first 24 months from dialysis initiation, there was a significant reduction in fatigue severity (beta = −0.15, *p* = .007, 95% CI −0.25 to −0.04); however, with a longer dialysis vintage (over 24 months), a small increase in fatigue severity, approaching significance, could be observed (beta = 0.01, *p* = .070, 95% CI −0.001 to 0.03; Supplementary File S2). In contrast, fatigue‐related functional impairment appeared relatively stable across dialysis vintage. In the first 24 months of dialysis vintage, there was a non‐significant increase in fatigue‐related functional impairment (beta = 0.17, *p* = .09, 95% CI −0.03 to 0.37), yet with a longer dialysis vintage (over 24 months), this increase flattened (beta = 0.01, *p* = .53, 95% CI −0.02 to 0.04; Supplementary File S3).

## Discussion

The aim of this paper was to assess the trajectories of fatigue outcomes over 3 years and to identify predictors of these among prevalent (those receiving dialysis for 3 months or longer) in‐centre HD patients.

Similar to previous estimates of the prevalence of fatigue among dialysis patients (Almutary *et al.*, 2013), we found that approximately one in two patients (47.1%) could be deemed clinically fatigued at baseline, and many of those fatigued at baseline, who were followed up, remained fatigued at each subsequent follow‐up.

The findings of the piecewise and linear growth models complement each other and reveal important nuances regarding fatigue change, suggesting that fatigue severity does not follow one linear trend of reduction over time. Fatigue severity appears to decrease within the first 24 months of dialysis, increasing thereafter with dialysis vintage.

While strong associations were evident across follow‐ups between all psychological predictors and fatigue outcomes, this was not reflected in the linear growth models, which is also in contrast to the cross‐sectional findings of this study (Chilcot *et al.*, 2016). Psychological factors did not appear to play a significant role in fatigue severity, after controlling for sociodemographic and clinical factors. A lower initial level of fatigue severity and a reduction in fatigue severity each year were predicted by non‐White ethnicity here. Contrary to expectations, an increase in haemoglobin was significantly associated with a small increase in fatigue severity each year. It is important to note that assessing the effects of time‐dependent haemoglobin using a single measurement per time‐point is less precise, and a time‐averaged haemoglobin estimate over 3 to 4 months would be more appropriate.

Similarly, none of the sociodemographic, clinical, nor psychological variables emerged as significant predictors of the initial level of fatigue‐related functional impairment. Damage beliefs in response to fatigue were the only significant predictor of an increase in fatigue‐related functional impairment each year. The stepped approach adopted with the linear models, revealed some other factors that may play a role in fatigue outcomes, including employment status, exercise status, and distress. However, these factors were attenuated by the addition of psychological predictors to the models, suggesting some collinearity between the variables.

The findings of this study make a valuable contribution to our understanding of fatigue outcomes over time among dialysis patients, exceeding follow‐up periods of previous longitudinal studies in this setting (Bossola *et al.*, 2017; Jhamb *et al.*, 2009, 2011). An important novel insight from this study is that fatigue is not merely associated with dialysis vintage, as previously suggested by some studies (Jhamb *et al.*, 2011), but rather changes over the course of dialysis vintage. In the longitudinal study of fatigue over 1 year among prevalent HD patients by Bossola *et al. *(2017), fatigue severity also appeared relatively stable and was generally unexplained by sociodemographic and clinical factors when using the arbitrary follow‐up time.

Contrary to expectations and previous findings on the important role beliefs and behaviours play in illness and symptom experience, including fatigue, across different LTCs (Hagger *et al.*, 2017), only damage beliefs in response to fatigue independently predicted greater fatigue‐related functional impairment over time here. The role of damage beliefs in fatigue‐related functional impairment was also evident in the cross‐sectional data (Chilcot *et al.*, 2016). Damage beliefs may be a proxy of actual damage occurring to the body as perceived by the patient; however, in line with existing literature, the majority of clinical factors, such as Hb, albumin, dialysis adequacy (URR), did not contribute significantly to fatigue severity and fatigue‐related functional impairment here and the association between damage beliefs and fatigue‐related functional impairment was above and beyond the role of clinical factors. Believing that fatigue is a sign of damage appeared particularly salient to renal patients and has been previously reported in a qualitative study, where patients often struggled to dissociate fatigue from the experience of the illness and treatment (Picariello *et al.*, 2017, 2017a). Higher levels of damage beliefs, alongside catastrophizing and symptom focusing, were also observed in a sample of fatigued RA patients, possibly maintaining and perpetuating fatigue for this group as well (Ali *et al.*, 2017).

In line with previous findings, non‐White patients experience lower fatigue and are less likely to experience an increase in fatigue over time in this patient population (Jhamb *et al.*, 2009, 2011; Kutner & Devins, 1998; Kutner *et al.*, 2000, 2005), possibly as a result of cultural differences in perception of the illness and treatment (Unruh *et al.*, 2004), variations in coping styles employed (Bhui *et al.*, 2011; Prelow *et al.*, 2000; Triandis *et al.*, 1984), or due to stigma associated with disclosing distress and maladjustment (Cooper‐Patrick *et al.*, 1997; de Crane & Spielberger, 1981; Thompson *et al.*, 2004). Education and health literacy are also likely to be important confounders here. Lower educational attainment and low health literacy may influence fatigue via a poorer understanding of the illness and non‐adherence, while patients with greater educational attainment may be more aware of the gravity and consequences of the illness. This further accentuates the complex aetiology of fatigue. It is also essential to consider this finding alongside the epidemiology of chronic kidney disease (CKD). The median age of patients on dialysis is highly dependent on ethnicity, specifically non‐White patients tend to be younger (Hole *et al.*, 2018; MacNeill *et al.*, 2018). Therefore, age is also a likely explanation here. It is also yet to be determined whether the self‐report instruments used here are cross‐culturally valid.

According to the findings here, the timing of intervention delivery may be of relevance for patients on dialysis, as 24 months from dialysis initiation marked a change in the trajectory of fatigue severity. There is accumulating evidence in support of psychological interventions, such as cognitive‐behavioural therapy for the management of fatigue in physical LTCs (van den Akker *et al.*, 2016) and promising preliminary evidence is available in kidney failure (Picariello *et al.*, 2017). However, what modifiable cognitive and behavioural factors are associated with fatigue trajectories over the span of dialysis vintage is yet to be defined.

To our knowledge, this is the first study to assess the contribution of fatigue‐specific cognitive and behavioural factors to fatigue among in‐centre HD patients. An additional strength of the study is its longitudinal design, enabling us to evaluate the trajectories of fatigue severity and fatigue‐related functional impairment over 3 years and identify factors that may predict changes in fatigue, in an area that is largely dominated by cross‐sectional research.

However, the current study has several important limitations. Firstly, although the sample consisted of 174 patients at baseline, it is still limited in size, particularly given the longitudinal study design, restricting the complexity of the analysis that could be conducted. In the light of findings in other LTCs, it would be clinically valuable to explore whether different clusters of fatigue trajectories exist in this patient population, using latent class growth modelling (Jung & Wickrama, 2008; Nagin & Odgers, 2010). Although high levels of attrition are inevitable in this patient population, the findings need to be interpreted with caution.

While the longitudinal design played a critical role in determining that fatigue changes with dialysis vintage, as prevalent HD patients were recruited into the study, this considerably limited our ability to use the natural trajectory of fatigue based on dialysis vintage to identify predictors of fatigue. Future studies should assess predictors of fatigue outcomes longitudinally using a larger sample of incident dialysis patients, those new to dialysis. However, such a study may prove challenging to conduct in practice, as the number of patients starting HD at any one centre per year is limited (Hole *et al.*, 2018). Fatigue is also common in CKD patients who do not require RRT (Abdel‐Kader *et al.*, 2009); therefore, dialysis initiation may not necessarily reflect symptom onset.

Despite the longitudinal study design, predictor and outcomes variables captured individuals’ internal states simultaneously, consequently reciprocal causation remains a problem (Singer & Willett, 2003). To circumvent this, asymptomatic CKD patients could be recruited and followed up beyond dialysis initiation; however, unless in the context of a large prospective epidemiological study, this is unlikely to be feasible in practice.

Although collecting data on‐dialysis provided consistency and minimized patient burden, there is evidence of significant fluctuations in fatigue as a consequence of dialysis (Brys *et al.*, 2019); therefore, to capture the full complexity of fatigue in this patient population, combining average and momentary assessments would be essential and would enable the identification of fatigue predictors both at the group level and at the individual level. Within‐person methodology can also help to circumvent the limited target population of incident dialysis patients.

Due to concerns about patient burden, the role of important factors like subjective sleep quality, social support, and representation of the illness was not assessed here. Additionally, the use of the total BIPQ score precluded us here from exploring the role of individual fatigue perceptions.

Additional limitations, including the possibility of non‐response bias, generalizability of findings to non‐English‐speaking samples, and sporadic availability of CRP from clinical records, have been previously addressed elsewhere (Chilcot *et al.*, 2016; Picariello *et al.*, 2018).

### Conclusion

In conclusion, changes in fatigue outcomes over time may be more complex than originally anticipated, varying by the length of time on dialysis, as the data suggested here. This provides some indication of when treatment for fatigue may be effectively delivered, yet the targets of treatment need to be explored further using a sample of incident dialysis patients.

## Funding

This work was funded by a Biomedical Research Studentship to Federica Picariello (FP) from the National Institute for Health Research (NIHR) Biomedical Research Centre at South London and Maudsley NHS Foundation Trust and King's College London. RMM acknowledges the financial support of the Department of Health via the National Institute for Health Research (NIHR) Specialist Biomedical Research Centre for Mental Health award to the South London and Maudsley NHS Foundation Trust (SLaM) and the Institute of Psychiatry at King's College London. The views expressed in this article are those of the authors and not necessarily those of the NHS, the NIHR, or the Department of Health.

## Conflict of interest

We hereby declare that to our knowledge there are no conflicts of interest. This work has not been published previously in whole or part, except in abstract format.

## Ethical approval

The study was reviewed and received ethical approval from an NHS Research Ethics Committee (East Midlands‐Leicester NRES committee, reference number 14/EM/0037) and has received local Research and Development (R&D) approval. All participants provided written informed consent. The study adhered to the Declaration of Helsinki (1964) ethical standards.

## Supporting information


**Supplementary File S1** Dialysis vintage frequency distribution graph

**Supplementary File S2** Trajectory of fatigue severity using dialysis vintage as time

**Supplementary File S3** Trajectory of fatigue‐related functional impairment using dialysis vintage as time

**Supplementary File S4** Predictors of fatigue outcomes over time, including participants self‐identifying as White, Black African, Black Caribbean, or Black Other

## Data Availability

The data that support the findings of this study are available on request from the corresponding author. The data are not publicly available due to privacy or ethical restrictions.

## References

[bjhp12395-bib-0001] Abdel‐Kader, K. , Unruh, M. L. , & Weisbord, S. D. (2009). Symptom burden, depression, and quality of life in chronic and end‐stage kidney disease. Clinical Journal of the American Society of Nephrology, 4, 1057–1064. 10.2215/CJN.00430109 19423570 PMC2689883

[bjhp12395-bib-0002] Ali, S. , Matcham, F. , Irving, K. , & Chalder, T. (2017). Fatigue and psychosocial variables in autoimmune rheumatic disease and chronic fatigue syndrome: A cross‐sectional comparison. Journal of Psychosomatic Research, 92, 1–8. 10.1016/j.jpsychores.2016.11.002 27998507

[bjhp12395-bib-0003] Almutary, H. , Bonner, A. , & Douglas, C. (2013). Symptom burden in chronic kidney disease: A review of recent literature. Journal of Renal Care, 39, 140–150. 10.1111/j.1755-6686.2013.12022.x 23826803

[bjhp12395-bib-0004] Alsén, P. , Brink, E. , Persson, L.‐O. , Brändström, Y. , & Karlson, B. W. (2010). Illness perceptions after myocardial infarction: Relations to fatigue, emotional distress, and health‐related quality of life. Journal of Cardiovascular Nursing, 25, E1–E10. 10.1097/JCN.0b013e3181c6dcfd 20168186

[bjhp12395-bib-0005] Bhui, K. S. , Dinos, S. , & Morelli, M.‐L. (2011). Ethnicity and fatigue: Expressions of distress, causal attributions and coping. Sociology Mind, 1, 156–163. 10.4236/sm.2011.14020

[bjhp12395-bib-0006] Bossola, M. , Di Stasio, E. , Antocicco, M. , Panico, L. , Pepe, G. , & Tazza, L. (2015). Fatigue is associated with increased risk of mortality in patients on chronic hemodialysis. Nephron, 130, 113–118. 10.1159/000430827 26021737

[bjhp12395-bib-0007] Bossola, M. , Di Stasio, E. , Antocicco, M. , Pepe, G. , Marzetti, E. , & Vulpio, C. (2017). 1‐year course of fatigue in patients on chronic hemodialysis. International Urology and Nephrology, 49, 727–734. 10.1007/s11255-016-1496-4 28054167

[bjhp12395-bib-0008] Bossola, M. , Vulpio, C. , & Tazza, L. (2011). Fatigue in chronic dialysis patients. Seminars in Dialysis, 24, 550–555. 10.1111/j.1525-139X.2011.00956.x 21917000

[bjhp12395-bib-0009] Broadbent, E. , Petrie, K. J. , Main, J. , & Weinman, J. (2006). The Brief Illness Perception Questionnaire. Journal of Psychosomatic Research, 60, 631–637. 10.1016/j.jpsychores.2005.10.020 16731240

[bjhp12395-bib-0010] Bryk, A. S. , & Raudenbush, S. W. (1992). Hierarchical linear models for social and behavioral research: Applications and data analysis methods. Newbury Park, CA: SAGE.

[bjhp12395-bib-0011] Brys, A. D. , Lenaert, B. , Van Heugten, C. M. , Gambaro, G. , & Bossola, M. (2019). Exploring the diurnal course of fatigue in patients on hemodialysis treatment and its relation with depressive symptoms and classical conditioning. Journal of Pain and Symptom Management, 57(5), 890–898.e4. 10.1016/j.jpainsymman.2019.02.010 30776536

[bjhp12395-bib-0012] Byrne, C. , Caskey, F. , Castledine, C. , Davenport, A. , Dawnay, A. , Fraser, S.., … Williams, A. (2018). 20th annual report of the renal association. Bristol, UK: UK Renal Registry 10.1159/000490957

[bjhp12395-bib-0013] Cella, D. , Peterman, A. , Passik, S. , Jacobsen, P. , & Breitbart, W. (1998). Progress toward guidelines for the management of fatigue. Oncology (Williston Park, NY), 12, 369–377. 10028520

[bjhp12395-bib-0014] Chalder, T. , Berelowitz, G. , Pawlikowska, T. , Watts, L. , Wessely, S. , Wright, D. , & Wallace, E. (1993). Development of a fatigue scale. Journal of Psychosomatic Research, 37, 147–153. 10.1016/0022-3999(93)90081-P 8463991

[bjhp12395-bib-0015] Charlson, M. E. , Pompei, P. , Ales, K. L. , & MacKenzie, C. R. (1987). A new method of classifying prognostic comorbidity in longitudinal studies: Development and validation. Journal of Chronic Diseases, 40, 373–383. 10.1016/0021-9681(87)90171-8 3558716

[bjhp12395-bib-0016] Cheema, B. S. B. , & Singh, F. (2005). Exercise training in patients receiving maintenance hemodialysis: A systematic review of clinical trials. American Journal of Nephrology, 25, 352–364. 10.1159/000087184 16088076

[bjhp12395-bib-0017] Chilcot, J. , Moss‐Morris, R. , Artom, M. , Harden, L. , Picariello, F. , Hughes, H. , … Macdougall, I. C. (2016). Psychosocial and clinical correlates of fatigue in haemodialysis patients: The importance of patients’ illness cognitions and behaviours. International Journal of Behavioral Medicine, 23, 271–281. 10.1007/s12529-015-9525-8 26607452

[bjhp12395-bib-0018] Cooper‐Patrick, L. , Powe, N. R. , Jenckes, M. W. , Gonzales, J. J. , Levine, D. M. , & Ford, D. E. (1997). Identification of patient attitudes and preferences regarding treatment of depression. Journal of General Internal Medicine, 12, 431–438. 10.1046/j.1525-1497.1997.00075.x 9229282 PMC1497133

[bjhp12395-bib-0019] Cramp, F. , Hewlett, S. , Almeida, C. , Kirwan, J. R. , Choy, E. H. , Chalder, T. , … Christensen, R. (2013). Non‐pharmacological interventions for fatigue in rheumatoid arthritis. Cochrane Database Systematic Review, 8, CD008322 10.1002/14651858.CD008322.pub2 PMC11748118 23975674

[bjhp12395-bib-0020] de Crane, R. S. , & Spielberger, C. D. (1981). Attitudes of Hispanic, Black, and Caucasion university students toward mental illness. Hispanic Journal of Behavioral Sciences, 3, 241–253. 10.1177/073998638100300302

[bjhp12395-bib-0021] Dittner, A. J. , Wessely, S. C. , & Brown, R. G. (2004). The assessment of fatigue: A practical guide for clinicians and researchers. Journal of Psychosomatic Research, 56, 157–170. 10.1016/S0022-3999(03)00371-4 15016573

[bjhp12395-bib-0022] Donovan, K. A. , Small, B. J. , Andrykowski, M. A. , Munster, P. , & Jacobsen, P. B. (2007). Utility of a cognitive‐behavioral model to predict fatigue following breast cancer treatment. Health Psychology, 26, 464–472. 10.1037/0278-6133.26.4.464 17605566 PMC2383279

[bjhp12395-bib-0023] Evangelidis, N. , Tong, A. , Manns, B. , Hemmelgarn, B. , Wheeler, D. C. , Tugwell, P. , … Winkelmayer, W. C. (2017). Developing a set of core outcomes for trials in hemodialysis: An international Delphi survey. American Journal of Kidney Diseases, 70, 464–475. 10.1053/j.ajkd.2016.11.029 28238554

[bjhp12395-bib-0024] Graham, J. W. (2009). Missing data analysis: Making it work in the real world. Annual Review of Psychology, 60, 549–576. 10.1146/annurev.psych.58.110405.085530 18652544

[bjhp12395-bib-0025] Grayson, P. C. , Amudala, N. A. , Mcalear, C. A. , Leduc, R. L. , Shereff, D. , Richesson, R. , … Merkel, P. A. (2013). Illness perceptions and fatigue in systemic vasculitis. Arthritis Care & Research, 65, 1835–1843. 10.1146/annurev.psych.58.110405.085530 23861259 PMC3962511

[bjhp12395-bib-0026] Hagger, M. S. , Koch, S. , Chatzisarantis, N. L. , & Orbell, S. (2017). The common sense model of self‐regulation: Meta‐analysis and test of a process model. Psychological Bulletin, 143, 1117–1154. 10.1037/bul0000118 28805401

[bjhp12395-bib-0027] Hole, B. , Gilg, J. , Casula, A. , Methven, S. , & Castledine, C. (2018). UK Renal Replacement Therapy adult incidence in 2016: National and centre‐specific analyses. Nephron, 139, 13–46. 10.1159/000490959 29990997

[bjhp12395-bib-0028] Horigan, A. E. (2012). Fatigue in hemodialysis patients: A review of current knowledge. Journal of Pain and Symptom Management, 44, 715–724. 10.1159/000490959 22743156

[bjhp12395-bib-0029] Jhamb, M. , Argyropoulos, C. , Steel, J. L. , Plantinga, L. , Wu, A. W. , Fink, N. E. , … Unruh, M. L. (2009). Correlates and outcomes of fatigue among incident dialysis patients. Clinical Journal of the American Society of Nephrology, 4, 1779–1786. 10.2215/CJN.00190109 19808226 PMC2774952

[bjhp12395-bib-0030] Jhamb, M. , Pike, F. , Ramer, S. , Argyropoulos, C. , Steel, J. , Dew, M. A. , … Unruh, M. (2011). Impact of fatigue on outcomes in the hemodialysis (HEMO) study. American Journal of Nephrology, 33, 515–523. 10.1159/000328004 21555875 PMC4484241

[bjhp12395-bib-0031] Jopson, N. M. , & Moss‐Morris, R. (2003). The role of illness severity and illness representations in adjusting to multiple sclerosis. Journal of Psychosomatic Research, 54, 503–511. 10.1016/S0022-3999(02)00455-5 12781303

[bjhp12395-bib-0032] Jung, T. , & Wickrama, K. (2008). An introduction to latent class growth analysis and growth mixture modeling. Social and Personality Psychology Compass, 2(1), 302–317. 10.1111/j.1751-9004.2007.00054.x

[bjhp12395-bib-0033] Kangas, M. , Bovbjerg, D. H. , & Montgomery, G. H. (2008). Cancer‐related fatigue: A systematic and meta‐analytic review of non‐pharmacological therapies for cancer patients. Psychological Bulletin, 134, 700–741. 10.1037/a0012825 18729569

[bjhp12395-bib-0034] Kosmadakis, G. , Bevington, A. , Smith, A. , Clapp, E. , Viana, J. , Bishop, N. , & Feehally, J. (2010). Physical exercise in patients with severe kidney disease. Nephron Clinical Practice, 115(1), c7–c16. 10.1159/000286344 20173344

[bjhp12395-bib-0035] Koyama, H. , Fukuda, S. , Shoji, T. , Inaba, M. , Tsujimoto, Y. , Tabata, T. , … Nishizawa, Y. (2010). Fatigue is a predictor for cardiovascular outcomes in patients undergoing hemodialysis. Clinical Journal of the American Society of Nephrology, 5, 659–666. 10.2215/CJN.08151109 20185601 PMC2849696

[bjhp12395-bib-0036] Kutner, N. G. , Brogan, D. , Fielding, B. , & Hall, W. (2000). Black/white differences in health outcomes reported by older ESRD patients on chronic hemodialysis. Ethnicity & Disease, 10, 328–333. 11110348

[bjhp12395-bib-0037] Kutner, N. G. , & Devins, G. M. (1998). A comparison of the quality of life reported by elderly whites and elderly blacks on dialysis. Geriatric Nephrology and Urology, 8, 77–83. 10.1023/A:1008384814079 9893215

[bjhp12395-bib-0038] Kutner, N. G. , Zhang, R. , & Brogan, D. (2005). Race, gender, and incident dialysis patients’ reported health status and quality of life. Journal of the American Society of Nephrology, 16, 1440–1448. 10.1681/ASN.2004080639 15800127

[bjhp12395-bib-0039] MacNeill, S. J. , Ford, D. , Evans, K. , & Medcalf, J. F. (2018). UK Renal Registry 20th Annual Report: UK renal replacement therapy adult prevalence in 2016: National and centre‐specific analyses. Nephron, 139, 47–74. 10.1159/000490960 29990998

[bjhp12395-bib-0040] Mazza, G. L. , Enders, C. K. , & Ruehlman, L. S. (2015). Addressing item‐level missing data: A comparison of proration and full information maximum likelihood estimation. Multivariate Behavioral Research, 50, 504–519. 10.1080/00273171.2015.1068157 26610249 PMC4701045

[bjhp12395-bib-0041] Mundt, J. C. , Marks, I. M. , Shear, M. K. , & Greist, J. H. (2002). The Work and Social Adjustment Scale: A simple measure of impairment in functioning. British Journal of Psychiatry, 180, 461–464. 10.1192/bjp.180.5.461 11983645

[bjhp12395-bib-0042] Nagin, D. S. , & Odgers, C. L. (2010). Group‐based trajectory modeling in clinical research. Annual Review of Clinical Psychology, 6, 109–138. 10.1146/annurev.clinpsy.121208.131413 20192788

[bjhp12395-bib-0043] Nordin, Å. , Taft, C. , Lundgren‐Nilsson, Å. , & Dencker, A. (2016). Minimal important differences for fatigue patient reported outcome measures – A systematic review. BMC Medical Research Methodology, 16(1), 62 10.1186/s12874-016-0167-6 27387456 PMC4937582

[bjhp12395-bib-0044] Picariello, F. , Hudson, J. L. , Moss‐Morris, R. , Macdougall, I. C. , & Chilcot, J. (2017). Examining the efficacy of social‐psychological interventions for the management of fatigue in End‐Stage Kidney Disease (ESKD): A systematic review with meta‐analysis. Health Psychology Review, 11, 197–216. 10.1080/17437199.2017.1298045 28277013

[bjhp12395-bib-0045] Picariello, F. , Moss‐Morris, R. , Macdougall, I. C. , & Chilcot, J. (2016). Measuring fatigue in haemodialysis patients: The factor structure of the Chalder Fatigue Questionnaire (CFQ). Journal of Psychosomatic Research, 84, 81–83. 10.1016/j.jpsychores.2016.03.124 27095163

[bjhp12395-bib-0046] Picariello, F. , Moss‐Morris, R. , Macdougall, I. C. , & Chilcot, J. (2017a). “It’s when you’re not doing too much you feel tired”: A qualitative exploration of fatigue in End‐Stage Kidney Disease (ESKD). British Journal of Health Psychology, 23, 311–333. 10.1111/bjhp.12289 29280249 PMC5900909

[bjhp12395-bib-0047] Picariello, F. , Moss‐Morris, R. , Macdougall, I. C. , & Chilcot, J. (2017b). The role of psychological factors in fatigue among End‐Stage Kidney Disease patients: A critical review. Clinical Kidney Journal, 10(1), 79–88. 10.1093/ckj/sfw113 28638608 PMC5469558

[bjhp12395-bib-0048] Picariello, F. , Norton, S. , Moss‐Morris, R. , Macdougall, I. C. , & Chilcot, J. (2018). Fatigue in prevalent haemodialysis patients predicts all‐cause mortality and kidney transplantation. Annals of Behavioral Medicine, 53, 501–514. 10.1093/abm/kay061 30020399

[bjhp12395-bib-0049] Prelow, H. M. , Tein, J.‐Y. , Roosa, M. W. , & Wood, J. (2000). Do coping styles differ across sociocultural groups? The role of measurement equivalence in making this judgment. American Journal of Community Psychology, 28, 225–244. 10.1023/A:1005139318357 10836092

[bjhp12395-bib-0050] Rabe‐Hesketh, S. , & Skrondal, A. (2008). Multilevel and longitudinal modeling using Stata. Berkeley, CA: STATA Press.

[bjhp12395-bib-0051] Ream, E. , & Richardson, A. (1996). Fatigue: A concept analysis. International Journal of Nursing Studies, 33, 519–529. 10.1016/0020-7489(96)00004-1 8886902

[bjhp12395-bib-0052] Ryan, E. G. , Vitoratou, S. , Goldsmith, K. A. , & Chalder, T. J. P. (2018). Psychometric properties and factor structure of a long and shortened version of the cognitive and behavioural responses questionnaire. Psychosomatic Medicine, 80, 230–237. 10.1097/PSY.0000000000000536 29023262

[bjhp12395-bib-0053] Segura‐Ortí, E. (2010). Exercise in haemodialysis patients: A systematic review. Nefrologia, 30, 236–246. 10.3265/Nefrologia.pre2010.Jan.10229 20098466

[bjhp12395-bib-0054] Singer, J. , & Willett, J. (2003). Applied longitudinal data analysis. Oxford, UK: Oxford University Press.

[bjhp12395-bib-0055] StataCorp Longitudinal . (2005). Stata longitudinal/panel data: Reference manual (9th release). College Station, TX: Stata Press.

[bjhp12395-bib-0056] Sterne, J. A. , White, I. R. , Carlin, J. B. , Spratt, M. , Royston, P. , Kenward, M. G. , … Carpenter, J. R. (2009). Multiple imputation for missing data in epidemiological and clinical research: Potential and pitfalls. British Medical Journal, 338, b2393 10.1136/bmj.b2393 19564179 PMC2714692

[bjhp12395-bib-0057] Thompson, V. L. S. , Bazile, A. , & Akbar, M. (2004). African Americans' perceptions of psychotherapy and psychotherapists. Professional Psychology: Research and Practice, 35(1), 19–26. 10.1037/0735-7028.35.1.19

[bjhp12395-bib-0058] Torres‐Reyna, O. (2007). Panel data analysis: Fixed and random effects using Stata (v. 4.2). Retrieved from http://www.princeton.edu/%7Eotorres/Panel101.pdf.

[bjhp12395-bib-0059] Triandis, H. C. , Marin, G. , Lisansky, J. , & Betancourt, H. (1984). Simpatia as a cultural script of Hispanics. Journal of Personality and Social Psychology, 47, 1363–1375. 10.1037/0022-3514.47.6.1363

[bjhp12395-bib-0060] Unruh, M. L. , Miskulin, D. , Yan, G. , Hays, R. D. , Benz, R. , Kusek, J. W. , …the HEMO Study Group . (2004). Racial differences in health‐related quality of life among hemodialysis patients. Kidney International, 65, 1482–1491. 10.1111/j.1523-1755.2004.00529.x 15086492

[bjhp12395-bib-0061] van den Akker, L. , Beckerman, H. , Collette, E. H. , Eijssen, I. C. J. M. , Dekker, J. , & de Groot, V. (2016). Effectiveness of cognitive behavioral therapy for the treatment of fatigue in patients with multiple sclerosis: A systematic review and meta‐analysis. Journal of Psychosomatic Research, 90, 33–42. 10.1016/j.jpsychores.2016.09.002 27772557

[bjhp12395-bib-0062] Van Der Leeden, R. (1998). Multilevel analysis of longitudinal data. London, UK: SAGE.

[bjhp12395-bib-0063] Van der Werf, S. , Evers, A. , Jongen, P. J. , & Bleijenberg, G. (2003). The role of helplessness as mediator between neurological disability, emotional instability, experienced fatigue and depression in patients with multiple sclerosis. Multiple Sclerosis, 9(1), 89–94. 10.1191/1352458503ms854oa 12617274

[bjhp12395-bib-0064] van Kessel, K. , & Moss‐Morris, R. (2006). Understanding multiple sclerosis fatigue: A synthesis of biological and psychological factors. Journal of Psychosomatic Research, 61, 583–585. 10.1016/j.jpsychores.2006.03.006 17084134

[bjhp12395-bib-0065] Vercoulen, J. , Swanink, C. , Galama, J. , Fennis, J. , Jongen, P. , Hommes, O. , … Bleijenberg, G. (1998). The persistence of fatigue in chronic fatigue syndrome and multiple sclerosis: Development of a model. Journal of Psychosomatic Research, 45, 507–517. 10.1016/S0022-3999(98)00023-3 9859853

[bjhp12395-bib-0066] Wessely, S. , Sharpe, M. , & Hotopf, M. (1998). Chronic fatigue and its syndromes. Oxford, UK: Oxford University Press.

[bjhp12395-bib-0067] White, P. D. , Goldsmith, K. A. , Johnson, A. L. , Potts, L. , Walwyn, R. , DeCesare, J. C. , … Cox, D. (2011). Comparison of adaptive pacing therapy, cognitive behaviour therapy, graded exercise therapy, and specialist medical care for chronic fatigue syndrome (PACE): A randomised trial. The Lancet, 377(9768), 823–836. 10.1016/S0140-6736(11)60096-2 PMC3065633 21334061

[bjhp12395-bib-0068] Zahra, D. , Qureshi, A. , Henley, W. , Taylor, R. , Quinn, C. , Pooler, J. , … Byng, R. (2014). The work and social adjustment scale: Reliability, sensitivity and value. International Journal of Psychiatry in Clinical Practice, 18, 131–138. 10.3109/13651501.2014.894072 24527886

[bjhp12395-bib-0069] Zalai, D. , Sherman, M. , McShane, K. , Shapiro, C. M. , & Carney, C. E. (2015). The importance of fatigue cognitions in chronic hepatitis C infection. Journal of Psychosomatic Research, 78, 193–198. 10.1016/j.jpsychores.2014.11.011 25433976

[bjhp12395-bib-0070] Zigmond, A. S. , & Snaith, R. P. (1983). The hospital anxiety and depression scale. Acta Psychiatrica Scandinavica, 67, 361–370. 10.1111/j.1600-0447.1983.tb09716.x 6880820

