# Supplementary material for: A prospective study of fatigue trajectories among in‐centre haemodialysis patients
Source: Br J Health Psychol. 2019 Nov 19;25(1):61–88. doi: 10.1111/bjhp.12395 (PMC7004141; doi:10.1111/bjhp.12395)
Supplement: Supplementary file 1 — Supplementary File S1 Dialysis vintage frequency distribution graph [file BJHP-25-61-s001.docx]

**Supplementary File S1: Dialysis vintage frequency distribution graph**


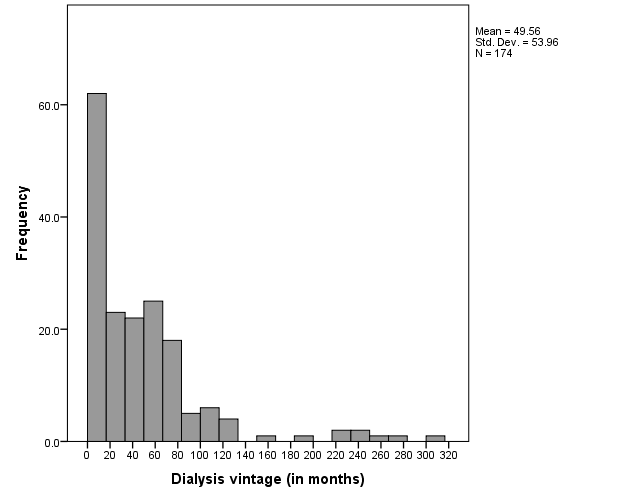


*Figure S1.* Histogram summarising the distribution of dialysis vintage across the sample at baseline.
