# Supplementary material for: A prospective study of fatigue trajectories among in‐centre haemodialysis patients
Source: Br J Health Psychol. 2019 Nov 19;25(1):61–88. doi: 10.1111/bjhp.12395 (PMC7004141; doi:10.1111/bjhp.12395)
Supplement: Supplementary file 2 — Supplementary File S2 Trajectory of fatigue severity using dialysis vintage as time [file BJHP-25-61-s002.docx]

**Supplementary File S2: Trajectory of fatigue severity using dialysis vintage as time**

*Figure S2.* Fatigue severity piecewise growth model using dialysis vintage as time (dialysis vintage displayed until 120 months)
