# Supplementary material for: A prospective study of fatigue trajectories among in‐centre haemodialysis patients
Source: Br J Health Psychol. 2019 Nov 19;25(1):61–88. doi: 10.1111/bjhp.12395 (PMC7004141; doi:10.1111/bjhp.12395)
Supplement: Supplementary file 3 — Supplementary File S3 Trajectory of fatigue‐related functional impairment using dialysis vintage as time [file BJHP-25-61-s003.docx]

**Supplementary File S3: Trajectory of fatigue-related functional impairment using dialysis vintage as time**

*Figure S3.* Fatigue-related functional impairment piecewise growth model using dialysis vintage as time (dialysis vintage displayed until 120 months).
