# Supplementary material for: A prospective study of fatigue trajectories among in‐centre haemodialysis patients
Source: Br J Health Psychol. 2019 Nov 19;25(1):61–88. doi: 10.1111/bjhp.12395 (PMC7004141; doi:10.1111/bjhp.12395)
Supplement: Supplementary file 4 — Supplementary File S4 Predictors of fatigue outcomes over time, including participants self‐identifying as White, Black African, Black Caribbean, or Black Other [file BJHP-25-61-s004.docx]

**Supplementary File S4: Predictors of fatigue outcomes over time, including participants self-identifying as White, Black African, Black Caribbean, or Black Other**

**Table S4.1**

*Predictors of Fatigue Severity (CFQ) Over Time, Including Participants Self-Identifying as White, Black African, Black Caribbean, or Black Other (N=156)*

|  | **Initial level of fatigue** | | | **Rate of change in fatigue** | | |
| --- | --- | --- | --- | --- | --- | --- |
| Step and variable | | *B* (95% CI) | Significance level (2-tailed) | Slope | *B* (95% CI) | Significance level (2-tailed) |
| Gender | | .80 (-1.25, 2.86) | 0.44 | 4.31 | -.17 (-1.16, .82) | 0.74 |
| Age (time-dependent) | | .004 (-.07, .07) | 0.91 |  | .005 (-.03, .04) | 0.79 |
| Ethnicity | | **-2.82 (-5.37, -.38)** | **0.024** |  | **1.36 (.34, 2.38)** | **0.009** |
| Employment status (at baseline) | | .003 (-2.78, 2.78) | 1.00 |  | -.15 (-1.46, 1.16) | 0.83 |
| BMI (at baseline) | | .04 (-.15, .24) | 0.67 |  | .002 (-.08, .08) | 0.96 |
| Exercise status (at baseline) | | -1.42 (-3.92, 1.08) | 0.26 |  | .38 (-.68, 1.43) | 0.48 |
| CCI (at baseline) | | .18 (-.46, .82) | 0.58 |  | -.03 (-.32, .26) | 0.84 |
| Dialysis vintage (time-dependent) | | .01 (-.01, .03) | 0.38 |  | .0008 (-.009, .01) | 0.86 |
| Haemoglobin (time-dependent) | | .07 (-.10, .24) | 0.40 |  | -.04 (-.09, .01) | 0.10 |
| Distress (time-dependent) | | .14 (-.23, .52) | 0.43 |  | .02 (-.09, .14) | 0.69 |
| Negative beliefs about fatigue (time-dependent) | | .07 (-.20, .35) | 0.59 |  | .00006 (-.06, .06) | 1.00 |
| Damage beliefs (time-dependent) | | .11 (-.65, .86) | 0.78 |  | -.07 (-.29, .15) | 0.51 |
| Embarrassment avoidance (time-dependent) | | .13 (-.37, .64) | 0.59 |  | -.08 (-.23, .07) | 0.27 |
| Avoidance behaviours (time-dependent) | | .12 (-.12, .36) | 0.31 |  | .009 (-.10, .11) | 0.87 |

**Table S4.2**

*Predictors of Fatigue-related Functional Impairment (WSAS) Over Time, Including Participants Self-Identifying as White, Black African, Black Caribbean, or Black Other (N=156)*

|  | **Initial level of fatigue** | | | **Rate of change in fatigue** | | |
| --- | --- | --- | --- | --- | --- | --- |
| Step and variable | | *B* (95% CI) | Significance level (2-tailed) | Slope | *B* (95% CI) | Significance level (2-tailed) |
| Gender | | -.06 (-3.38, 3.27) | 0.97 | 2.43 | .48 (-1.21, 2.18) | 0.58 |
| Age (time-dependent) | | -.0008 (-.13, .13) | 0.99 |  | -.003 (-.06, .07) | 0.93 |
| Employment status (at baseline) | | -3.79 (-8.63, 1.05) | 0.12 |  | .74 (-1.61, 3.10) | 0.54 |
| BMI (at baseline) | | .04 (-.27, .35) | 0.81 |  | .02 (-.13, .16) | 0.81 |
| Exercise status (at baseline) | | -1.03 (-4.92, 2.86) | 0.60 |  | .34 (-1.47, 2.16) | 0.71 |
| CCI (at baseline) | | .23 (-.74, 1.20) | 0.64 |  | .15 (-.33, .63) | 0.54 |
| Transplant list status (at baseline) | | 2.59 (-1.67, 6.85) | 0.23 |  | -1.26 (-3.29, .76) | 0.22 |
| Dialysis vintage (time-dependent) | | .02 (-.02, .07) | 0.32 |  | -.01 (-.03, .005) | 0.17 |
| Haemoglobin (time-dependent) | | -.11 (-.34, .13) | 0.36 |  | .03 (-.06, .12) | 0.48 |
| Distress (time-dependent) | | .15 (-.58, .88) | 0.36 |  | .01 (-.20, .22) | 0.92 |
| Negative beliefs about fatigue (time-dependent) | | .12 (-.29, .54) | 0.54 |  | -.02 (-.14, .10) | 0.77 |
| Damage beliefs (time-dependent) | | .60 (-.35, 1.55) | 0.21 |  | -.30 (-.62, .02) | 0.068 |
| Embarrassment avoidance (time-dependent) | | .08 (-.83, .98) | 0.86 |  | .10 (-.15, .35) | 0.42 |
| Avoidance behaviours (time-dependent) | | .31 (-.44, 1.05) | 0.40 |  | -.05 (-.24, .13) | 0.58 |
| Fatigue severity (time-dependent) | | .58 (-.15, 1.31) | 0.11 |  | -.07 (-.29, .14) | 0.49 |
